# Household recovery in Mosul one year after the defeat of ISIS

**DOI:** 10.1186/s13031-019-0247-4

**Published:** 2020-01-03

**Authors:** R. Lafta, M. Al-Nuaimi, L. R. Sultan, G. Burnham

**Affiliations:** 1 Department of Family and Community Medicine, Al Mustansiriya University, Baghdad, Iraq; 2 Department of International Health, The Johns Hopkins Bloomberg School of Public Health, 615 N Wolfe Street, Baltimore, Baltimore, MD MD, 21205 USA

**Keywords:** Mosul, Iraq, War, ISIS, Recovery

## Abstract

**Background:**

Widespread devastation to structures and households in Mosul occurred during the three years of ISIS control and the military liberation campaign by Iraqi forces assisted by coalition forces. Military operations, particularly airstrikes, resulted in a greater loss of life than during ISIS control. In 2016/17, we assessed living circumstances in Mosul immediately following defeat of ISIS. In September 2018, we reassessed many of the same indicators in Mosul households to determine the extent of recovery.

**Methods:**

For the 2018 survey, a random selection of 20 clusters were drawn from the 40 clusters surveyed in 2016/17. Of these 20 clusters, 12 were in east Mosul and 8 in west Mosul, the same proportion as the original survey. In each cluster, 30 households were interviewed. No households were included in both surveys. A team of four interviewers collected information using questions adapted from the 2016/17 questionnaire.

**Results:**

Among the 3375 persons from the 600 households in the 2018 survey, there had been 18 deaths reported in the year since the end of ISIS control, a mortality rate of 6.1/1000 (CI_95%_ [2.4–9.8]). This compares with a mortality rate of 30.7/1000 (CI_95%_ [28.3–33.2]) during ISIS control and liberation. Fifteen deaths were from disease, one from a non-intentional injury and two deaths due to intentional violence. Damage to dwellings had been fully repaired in only 22 (5.5%) of houses, mostly in less damaged east Mosul. Dramatic improvements in access to water and electricity have occurred, with three quarters of households reporting uninterrupted access to both. The previously reported large number of early marriages among household members stopped with the departure of ISIS. Of the 31 household marriages reported over a 12-month follow on study, 6 (19.4%) involved a female member of the household. This compares with 131 household males and 688 household females married during ISIS occupation. If marriages had continued at the same rate as for ISIS years during our one-year follow-on study, there would have been and expected 24 marriages of household males and 126 marriages of household females (OD 32.8, CI_95%_[10.5102.8]) *p <* 0.001. There were 657 children reported by households to be in primary school. However, by household listing there were only 380 of children in the usual primary school age range (6–11), suggesting older children are catching up on primary schooling missed during ISIS years. One report of physical violence between spouses occurred. By comparison, the adjusted number of reported violent spousal events during ISIS control and military action would have been 72.7 (OR 316.7, CI_95%_ [44.42259.9]), *p <* 0.001. Reported complications of pregnancy also declined (OR 10.3, CI_95%_ [5.4,19.4], *p* < 0.001.

**Conclusions:**

Substantial improvements in household measures have occurred since the end of ISIS control and military action, though much remains for full recovery. Many household members are now employed, primary school attendance is high and early marriage of girls was not found. There are fewer reported complications of pregnancy than in the previous study.

## Background

Mosul was occupied by ISIS in June 2014, causing an estimated one million people to flee to Kurdistan or neighboring governorates [1]. For those who remained there were three years of strict ISIS control during which severe punishment was meted out for the smallest infraction of the rigid behavior codes [2]. Many residents fled at the beginning, but most remained. The ISIS control ended with the devastating military campaign known as the “liberation,” which started in east Mosul in October 2016. During military operations there was widespread civilian death and destruction [3]. On the 10th of July 2017, Iraqi Prime Minister Al-Abadi formally announced the liberation of Mosul. While the damage to east Mosul was extensive, west Mosul, the old city, took the brunt of the devastating military assault. Many neighborhoods were still largely uninhabited [3]. An estimated 7.6 million tons of debris remain in Mosul, much contaminated with unexploded ordnance and booby traps [4]. During 2018, some 17,000 explosive devices were removed, with unknown numbers remaining [5]. Massive challenges remain in restoring Mosul and returning populations displaced in the conflict [6, 7].

Early international efforts have addressed the reconstruction of Mosul with pledges of US$30 billion. But this amount is only a third of estimated needs, and many pledges as loans rather than grants [8]. The European Union (EU) provided an additional € 57.5 million for Mosul in December, 2018 [9]. Initial efforts focused on returning displaced populations, clearing mines and rubble, and restoring utilities [10, 11]. By late 2018, there were still 325,596 persons displaced in neighboring Duhok, and 141,060 displaced elsewhere in the Ninawa governorate, of which Mosul is a part [12]. Yet some persons fear to return or have little of their former lives left in Mosul to reclaim [13]. Efforts to restart businesses are hampered by the extensive destruction of the ISIS years and liberation [14].

In 2017, immediately following cessation of fighting, we conducted a study of population health status of Mosul using a sample of 1202 households [15]. During the three years of ISIS control there was a substantial impact on household behavior. Access to health services was difficult, children were not allowed to attend school for fear of exposure to radical messages, and employment was difficult. Early marriage was common, arising from fears of forced marriage of Mosul residents to foreign fighters. During the liberation period there were far more deaths among Mosul residents than occurred during the ISIS years, especially in west Mosul [3]. Most of the damage and death was caused by air strikes. Of 54 residential neighborhoods in west Mosul, 38 had heavy or moderate damage, particularly in west Mosul [7]. Electricity was largely absent, and most water came from improvised wells. At the end of the military actions, there was widespread unemployment and under employment in the households sampled in 2016/7.

## Methods

To understand how the lives of Mosul residents have changed in the year following the liberation from ISIS control, we conducted this household survey in September 2018. We started with the same 40 clusters identified for the 2016/7 Mosul household survey [15]. For that survey, the residential administrative units or neighborhoods of Mosul were listed. These residential administrative units would commonly contain around 400 households. From this listing, 40 neighborhoods were selected as clusters, 15 in west Mosul and 25 in east Mosul, a ratio approximating the pre-ISIS population distribution of Mosul. In each cluster, a random point was selected using satellite imagery. The survey began with the household nearest to the random start point and moved to the nearest subsequent households until 30 households had been interviewed. A household was defined as a group of people living together, eating from a common kitchen and living in a structure with a separate entrance from the street. The sampling process is described more fully elsewhere [3]. For the one-year follow up survey reported here, 30 households were selected from each of 12 clusters randomly chosen from the original 25 in east Mosul, and from eight clusters of the previous 15 west Mosul clusters. A total of 600 households from 20 clusters were interviewed in September 2018. No households included in the 2016/17 survey were sampled again in this 2018 follow-on survey. Data from completed forms were computer entered in SPSS in Baghdad and preliminary analysis conducted there. Further analysis using Stata^1^ was conducted in Baltimore.

The questionnaire was adapted from the 2016/17 survey. Interviewers began with a household listing of persons in the household on or after July 2017. Demographic and educational characteristics were collected including births, deaths, injuries and marriages. Household and economic characteristics were recorded, as well as employment and economic indicators.

The interview team was composed of four persons with doctoral degrees in community medicine and who had extensive experience in community surveys. All were originally from Mosul. Some interviewers had participated in the 2016/17 survey. Two days of training was provided.

## Results

### Demographic characteristics

There were 600 households included in the survey, 360 in east Mosul and 240 in west Mosul. All households had been continuously present in Mosul since the liberation in July 2017. Only 24 households (4%) had not been present in Mosul before July 2017. In the 600 households there were 3375 persons, with a mean household size of 5.6 (CI_95%_[5.53, 5.68]) persons. Households were larger in in west Mosul than east (6.2 vs. 5.2, *p <* 0.001). Children under age 15 years constituted 29.6% of household members, 26.0% in east Mosul and 34.1% in west Mosul. The differenceOnly three households declined to participate in the survey. The household demographic characteristics are shown in Table 1.

**Table 1:** Demographic characteristics of surveyed households

	East Mosul (%)		West Mosul (%)		All Mosul (%)	
	Male	Female	Male	Female	Male	Female
Young children (0–4 years)	104 (10.5)	61 (5.8)	70 (9.6)	64 (8.3)	174 (10.1)	125 (7.6)
Older children (5–14 years)	154 (15.6)	135 (15.3)	164 (22.4)	169 (21.9)	318 (18.5)	304 (18.4)
Adults (15–49 years)	525 (53.1)	516 (58.4)	371 (50.7)	402 (52.2)	896 (52.1)	918 (55.5)
Older adults (50+ years)	206 (20.8)	172 (19.5)	127 (17.3)	135 (17.5)	333 (19.3)	307 (18.6)
Total	989	884	732	770	1721	1654
Mean household size	5.2		6.2		5.6	

### Education

Among household members, excluding those pre-school age or in school, there were only 166 women (13.5%) and 36 males (3.1%) who had no formal education. Among women, 336 (27.3%) had a primary school education, 298 (24.2%) had a secondary education and 35.1% had completed a post-secondary education. Among males, 287 (25.0%) had only primary school 284 (24.7%) had completed secondary school and 540 (47.1%) had a post-secondary education.

In the household listings there were 622 children in the 5–14-year-old age range and 380 children in the 6–11-year age group, the usual age group for primary school. However, the number of children currently reported by households to be in primary school was 657(data not shown).

### Employment

Excluding the pre-school children and students in the household, 745 (19.4%) persons reported working full time, and 674 (17.5%) worked part time (Table 2). There were 787 (20.4%) persons who listed their occupation as housewife, and there were 103 persons (2.7%) who reported that they were unemployed. Among the household’s residents there were 216 (5.6%) who were reported as retired. Overall, 61.5% of the adults who were of working age and not retired were employed outside the home in income producing activities. The mean household income for east Mosul was IQD 576,213 (CI_95%_ [576,168, 576,258])($461) and for west Mosul IQD 419,820 (CI_95%_ [419,783, 419,857])($366), *p* < 0.001.

**Table 2:** Employment status

	East Mosul		West Mosul		All Mosul	
	Male (%)	Female (%)	Male (%)	Female (%)	Male (%)	Female (%)
Work full time	237 (27.6)	387 (23.2)	90 (14.0)	31 (4.5)	327 (21.8)	418 (17.8)
Work part time	201 (23.4)	208 (12.5)	255 (39.8)	10 (4.5)	456 (30.4)	218 (9.3)
Unemployed	26 (3.0)	35 (2.1)	41 (6.4)	1 (0.1)	67 (4.5)	36 (1.5)
Student	313 (36.4)	579 (34.7)	220 (34.3)	213 (31.2)	533 (35.5)	792 (33.7)
Housewife	0	365 (21.9)	0	422 (61.9)	0	787 (33.5)
Retired	83 (9.7)	93 (5.6)	35 (5.5)	5 (0.7)	118 (7.9)	98 (4.2)
Mean monthly income, HH members working full time, Iraq dinars	958,416 (US$767)		718,545 ($575)			
Mean monthly income, HH members working part time, Iraq dinars	379,544 ($310)		219.271 ($175)			
Mean household income, all households, Iraq dinars	576,213 ($461)		419,820 ($366)			

### Injuries and death

Between July 2017 and September 2018, there were 28 injuries reported and 18 deaths among the 600 households (Table 3). There were three infant deaths. Among the non-fatal injuries, only six of the 28 were admitted to hospital. Four were treated at home and the remainder were treated as clinic outpatients. There were two intentional violence-related deaths. One person stepped on an explosive in the backyard of a house and a second returned to his vacant house, and later was found dead from an explosive injury. Mean ages for injuries was 42.9 years, and age for adult deaths was 48.9 years.

**Table 3:** Deaths and Injuries since July 2017

Death and Injury	East Mosul		West Mosul		All Mosul		Totals
	Male	Female	Male	Female	Male	Female	
Deaths since July 2017	2	3	2	11	4	14	18
Deaths from intentional violence	1	1	0	0	1	1	2
Deaths from injury	1	0	0	0	1	0	1
Deaths from disease	1	1	2	11	3	12	15
Causes of death from disease							
Diarrhea	0	0	0	0	0	0	0
Respiratory disease	1	0	2	0	3	0	3
Cancer/Tumor	0	1	0	4	0	5	5
Cardiovascular disease	0	0	0	4	0	4	4
Others	0	0	0	3	0	3	3
Injuries							
All Injuries since July 2017	15	4	5	4	20	8	28
Non-intentional violence injuries	15	4	5	1	20	5	25
Treatment at the time of Non intentional injury							
Home treatment	3	1	0	0	3	1	4
Treated in surgery or clinic (outpatient)	6	0	2	4	8	4	12
Admitted to hospital (inpatient)	6	0	3	0	9	0	9
No treatment	0	0	0	0	0	0	0
All Injuries							
Burn	2	0	1	0	3	0	3
Fall	5	0	0	0	5	0	5
Mechanical	4	4	4	0	8	4	12
Explosion	3	0	0	1	3	1	4
Intentional violence	0	0	0	3	0	3	3
Other	1	0	0	0	1	0	1
Mean age at time of injury (yrs.)	39.5	58	26.6	61.8	36.3	59.9	
Current status of injured							
Alive, functioning normally	12	4	3	0	15	4	19
Alive, with reduced function	2	0	0	3	2	3	5
Dead	1	0	0	0	1	0	1
Still under treatment	1	0	2	`1	3	1	4

### Housing and utilities

Damage to dwellings during ISIS years and the liberation was extensive, particularly in west Mosul. Some repairs have since occurred, but only 22 (5.5%) houses have been fully repaired (Table 4). No repairs have taken place in 41 (10.3%) houses, primarily in west Mosul. Most household reported continuous electricity, usually with private neighborhood generators supplementing the grid. Water was now present all the time in three-quarters of houses interviewed. Almost no houses reported lack of any access to water or electricity.

**Table 4:** Housing status and utilities

	East Mosul 360	West Mosul 240	All Mosul 600
House repairs (%)			
House damaged before July 2017	215 (59.7)	184 (76.7)	399 (66.6)
House now fully repaired^a^	21 (9.8)	1 (0.5)	22 (5.5)
House partially repaired^a^	183 (85.1)	153 (83.1)	336 (84.2)
No repairs^a^	11 (5.1)	30 (16.3)	41 (10.3)
Presence of electricity (%)			
All the time	269 (74.7)	186 (77.5)	455 (75.8)
Sometimes	90 (25.0)	50 (20.8)	140 (23.3)
Seldom or never	1 (0.2)	4 (1.7)	5 (0.8)
Access to water (%)			
All the time	258 (71.7)	201 (83.8)	459 (76.5)
Sometimes	101 (28.1)	38 (15.8)	139 (23.2)
Seldom or never	1 (0.2)	1 (0.4)	2 (0.3)

^a^of those damaged

### Marriage

Among the 600 households, there were 31 marriages in the past of year, of which 26 were of male members of the household. The mean age of marriage for males was 26.7 (range 23–31) years and for the five women was 25.4 years (range18–28). The youngest marriages reported were at age 18.

### Domestic violence

There were 42 reports of interpersonal violence within the family. Only three of these were instances of physical violence, the remainder being verbal. One physical abuse report involved a spouse and the other reports were among family members. In 10 instances there was abuse of a household member which was perpetrated by a family member resident outside the household.

### Comparisons with the 2016/17 household survey

#### Household size

In the 2016/17 mean household size was 6.29 (CI_95%_[6.25,6.23]) persons, and in the 2018 survey mean household size was 5.6 (CI_95%_ [5.39,6.81]) persons, *p <* 0.001. The proportion of adults present in household in 2016/17 was 60.3% and in 2018 it was 72.7%, *p <* 0.001.

#### Age of marriage

In the 2016/17 study there were 688 marriages among women in study households (range 11–26 years). Of these 253 of the 688 marriages were among women aged 18 or less (36.8%) and 89 of 688 were aged 15 or less (12.9%). For the 2018 survey, there were six household marriages among women, the youngest being two women aged 18. Mean age of marriage of a household member (male or female) during ISIS years was 20.4 years (median 20) and during the this one-year follow-on survey 26.4 years (median 26), *p <* 0.001.

#### Ratio of household marriages

Of the 31 household marriages reported over a 12-month follow on study, 6 (19.4%) involved a female member of the household. This compares with 131 household males and 688 household females married during ISIS occupation. If marriages had continued at the same rate as for ISIS years during our one-year follow-on study, there would have been an expected 24 marriages of household males and 126 marriages of household females (OR 32.8, CI_95%_ [10.5, 102.8]) *p <* 0.00.1.

#### Domestic violence

During years of ISIS control and the period of liberation there had been 415 reported cases of physical violence between husband and wife leading to injury of the wife. In the one-year follow-on survey, there had been only one reported instance of physical violence between husband and wife resulting in an injury.

#### Mortality

All-cause mortality during ISIS and the subsequent liberation was 30.7/1000/year (CI_95%_ [28.3,33.2]) which compares with a mortality rate of 6.1/1000/year (CI_95%_[2.4,9.8]) during the one-year post conflict follow-on survey.

#### Obstetrical complications

During ISIS years and the liberation there were 640 deliveries of which 417 were reported to have complications. In the year following liberation there were 12 complications reported among 78 deliveries, (OR 10.3 CI_95%_[5.4.19.4]), *p* < 0.001).

## Discussion

This study has allowed the assessment of recovery at the household level a year after the end of one of the most intense urban conflicts in decades [16]. Since the military defeat of ISIS in Mosul in early 2017, the households of Iraq’s second city have experienced an uneven recovery. Many persons, whose Mosul houses were not destroyed, have returned to Mosul from displacement in camps or from informal accommodation. Parts of Mosul, such as shops and small businesses, have seen rapid recovery. Yet there are many heavily damaged and barely inhabited areas in west Mosul [17]. Large industries with many employees have not recovered, as most industrial sites were looted or vandalized by ISIS or destroyed in the liberation [14]. Health services were badly damaged, particularly at the secondary and tertiary levels [15]. The housing needs for displaced persons in all Iraq are the highest in the Mosul area [17]. In various parts of the city there has been a sense of working together for community recovery, in the absence of much external assistance [18]. UNESCO has supported cooperative efforts which have helped rebuild the spirit of the city [19]. In the perceptions of the Mosul population, the assistance being provided comes from local NGOs, informal associations, and from various bilateral and multilateral donors, with little assistance from the local or central government [20]. However, government salaries have been largely restored, bringing money into the community.

### Household composition

When the household composition in this one-year follow-on survey was compared to households at the end of ISIS control, changes are noted. In this one-year repeat survey, household size in east Mosul had dropped from 6.5 to 5.2 persons while that in west Mosul had risen slightly from 6.0 to 6.2 person. It is likely this represents both return and redistribution of people following the conflict.

### Education

Only 2.2% of primary school age children attended school during years of ISIS control, reportedly because of family fear of exposure of children to radical ISIS teachings [15]. The current survey found 657 children who were reported to be in primary school, 58% greater than the number of primary school aged children in the survey households. This suggests that older children may now be in primary school, catching up with years of primary school missed during ISIS years. Primary school is compulsory in Iraq.

### Employment

In the current survey, 61.5% of adult household members of working age had full-time or part-time employment outside the household. The proportion of males working full time in west Mosul was about half of that of males in east Mosul households. This is consistent with the reported movement of able-bodied males from west Mosul following the devastating bombardment, and an indicator that employment has returned more rapidly in east Mosul. The fewer number of household members retired in west Mosul (3.0%) compared with east Mosul (6.9%), is consistent with the poverty of west Mosul, as retirement income is an important source of household income. The number of people reporting being unemployed was very small. In comparison, our survey immediately following the liberation found the largest single employment status for heads of households was “no work,” (29.4%) [15]. Mean household income was IQD 576,213 in east Mosul (US$461) and IQD 419,820 ($366) for west Mosul. By comparison in adjacent Kurdistan in 2018, half of households had a monthly income of between IQD 500,000 (US$400) and IQD 1,000,000 (US$800) [21].

### Injury and death

Although the sample size is small, the change in causes of death from the previous Mosul survey is striking. The all-cause mortality rate for this survey was 6.1/1000 (CI_95%_[2.4,9.8]). This compares with current estimated Iraq death rate of 4/1000 [22]. and death rate during ISIS and liberation of 30.7/1000 (CI_95%_[28.3,33.2] [15]. Of the 18 deaths since July 2017, only two were related to violence, and both from unexploded ordnance. Deaths from other causes, including deaths from cancers and cardiovascular conditions and from injuries due to unintentional violence, were similar to our findings immediately at the end of ISIS control [15]. By comparison, in our study of deaths during the years of ISIS control and liberation there were 628 deaths reported, of which 505 were due to intentional violence, most occurring during the military liberation campaign.

The massive amount of war debris mixed unexploded ordnance creates a great risk of injuries in Mosul. Injuries reported by households related to falls, burns, mechanical injuries and three from intentional violence, predominantly among males. The injury pattern was similar to previous reports from Baghdad, though motor vehicle injuries were absent in Mosul, probably from lack road traffic [23]. The mean age of injury for women was 59.9 years, 23.6 years older than injured men. By contrast, few injuries of any type were reported in the previous survey during the period of ISIS control.

### Marriage

In this follow up survey, there were 31 marriages reported for a member of the household 83.2%. were by a male member of the household. Mean ages were in in the twenties, with the youngest marriages reported at age 18. During the time of ISIS control, 84% of marriages by households had been among female members, with about half occurring in persons aged 18 and under, and 13% aged 15 or less [15]. Applying numbers from the ISIS years to the first year post ISIS control there would have been 124 marriages, and 84% household females among a comparable number of households. This finding is consistent with the interpretation that early marriage of girls during ISIS arose from a household fear of forced marriage of Mosul females to ISIS fighters, a situation seen in other conflict situations as well [24].

### Shelter

Housing repairs and the presence of electricity and domestic access to water are important indicators of recovery [14]. In the immediate post ISIS survey, we noted that overall 74.2% of the 1202 dwellings had some conflict damage, but in west Mosul 449 of 451 (99.6%) households surveyed had damage [15]. In this one-year follow-on survey, we found that overall only 5.5% of the 399 damaged houses had been fully repaired. Housing repairs were the least in west Mosul. These findings of extensive housing damage unrepaired is consistent with the 2019 IOM assessment [14]. Areas of historically high poverty levels sustained the greatest conflict damage, a finding for west Mosul in our 2016/17 survey. Overall, community recovery is linked with the reconstruction of dwellings, and dwelling reconstruction becomes less likely when living conditions are poor [14]. Water is now widely present at the household without the reliance on temporary hand dug wells. Electricity, which was almost totally absent at the time of liberation, is now reported in all houses. A quarter reported electricity as intermittent and these may have been houses supplied by local or neighborhood generators. Repair of utilities has been an early initiative for Mosul [25].

### Interpersonal violence

Over the 12-month period, there were 42 reports of interpersonal violence, verbal or physical among family members, likely a substantial undercounting. Only one reported instance of physical violence was between spouses. Apply rates from ISIS years there would have been 72.7 events. In a review of literature, Rubenstein and colleagues found that common predictors of interpersonal violence in the household in humanitarian settings included political and conflict exposure, poor economic status, mental health issues, limited coping strategies, and limited social support [26]. Political violence was also associated with violence toward women and children. During ISIS control and the liberation, many of these factors may certainly occured. Increasing employment and early urban recovery may have contributed to decreased interpersonal violence in comparison to the reports immediately post conflict.

### Limitation

A study of this nature has limitations. Households were sampled from clusters previously selected on a geo-spatial basis, in the absence of population data for Mosul. The population return and redistribution of existing populations is likely to be uneven across the sample sites. The sample was taken from the same clusters as previously (though not the same households), to make it comparable with our 2016/17 survey. The recovery of Mosul is unlikely to be similar for all areas, so these clusters may not be fully representative of all parts of Mosul. Some heavily damaged and minimally populated areas of west Mosul were not included. There were 24 of the 600 households which had not been present during ISIS occupation, but had been present for the past year. These may have had responses differing from the households present during the ISIS times, thus introducing some bias. While all households were present in Mosul for the past year, it is very likely that several household members would have returned more recently from temporary locations to which they fled. This could change some factors from the earlier study such as household composition. These household members may have spent their time away from Mosul in locations where the risk of injuries and death were less. Some of the survey questions referred to sensitive issues which may not have been fully reported. We had no independent method of verifying reported wages. Despite the limitations, we believe that resampling from the same 2016/7 cohort provides a valuable benchmark for measuring the recovery from the years of ISIS and the military liberation.

## Conclusion

Since the expulsion of ISIS, many elements of normal life have returned to Mosul. There is extensive employment, utilities are present at least some of the time. Repairs to the extensive housing damage has been slow. The high rate of female marriage previously noted, has now disappeared. Domestic violence between spouses has diminished. Intentional violence is not now a major cause of injury and death. Collapsed buildings and unexploded ordnance remain major risks to the Mosul population. The recovery of Mosul life is just beginning. A strong economic and employment environment with extensive international assistance will be needed to achieve a full recovery.

## Data Availability

The data set and materials reported in this study are available from the corresponding author on reasonable requests.

## Footnotes

1 Stata Corp. College Station, Texas.
